# Predictive modeling of COVID-19 death cases in Pakistan

**DOI:** 10.1016/j.idm.2020.10.011

**Published:** 2020-11-07

**Authors:** Muhammad Daniyal, Roseline Oluwaseun Ogundokun, Khadijah Abid, Muhammad Danyal Khan, Opeyemi Eyitayo Ogundokun

**Affiliations:** a Department of Statistics, Islamia University of Bahawalpur, Pakistan; b Department of Computer Science, Landmark University Omu Aran, Nigeria; c Research Evaluation Unit, College of Physicians & Surgeons, Pakistan; d Gillani Law College, BZU Multan, Pakistan; e Directoriate Department, Audit Section, Agricultural and Rural Management Training Institute, Ilorin, Nigeria

**Keywords:** Coronavirus, COVID-19, Public health, Epidemic, Modelling, Model selection criteria

## Abstract

**Background:**

The world is presently facing the challenges posed by COVID-19 (2019-nCoV), especially in the public health sector, and these challenges are dangerous to both health and life. The disease results in an acute respiratory infection that may result in pain and death. In Pakistan, the disease curve shows a vertical trend by almost 256K established cases of the diseases and 6035 documented death cases till August 5, 2020.

**Objective:**

The primary purpose of this study is to provide the statistical model to predict the trend of COVID-19 death cases in Pakistan. The age and gender of COVID-19 victims were represented using a descriptive study.

**Method:**

ology: Three regression models, which include Linear, logarithmic, and quadratic, were employed in this study for the modelling of COVID-19 death cases in Pakistan. These three models were compared based on R^2^, Adjusted R^2^, AIC, and BIC criterions. The data utilized for the modelling was obtained from the National Institute of Health of Pakistan from February 26, 2020 to August 5, 2020.

**Conclusion:**

The finding deduced after the prediction modelling is that the rate of mortality would decrease by the end of October. The total number of deaths will reach its maximum point; then, it will gradually decrease. This indicates that the curve of total deaths will continue to be flat, i.e., it will shift to be constant, which is also the upper bound of the underlying function of absolute death.

## 1 Introduction

The COVID-19 pandemic has emerged very rapidly worldwide, affecting nearly 5,488,825 individuals with 349,095 deaths (WHO, 2020a, WHO, 2020b). Initially, COVID-19 was thought to be a zoonotic virus (bat to human transmission); however, recent studies and the exponential increases in the incidence of COVID-19 indicate complete evidence of transmission from person to person (Adeniyi et al., 2020; Li et al., 2020a; Ogundokun and Awotunde, 2020; WHO, 2020a, WHO, 2020b). The first human exposure case was connected to a “wet market” from Wuhan, Hubei Province, China, in late December 2019 (Abid et al., 2020; Lu et al., 2020; Lukman et al., 2020; Ogundokun, Lukman, Kibria, Awotunde, & Aladeitan, 2020a, 2020b). The source of transmission was via droplets when an individual infected cough; it then entered into the human body and caused deteriorating effects on the intestines, spleen, and lungs. Even a single cough of corona infected individuals can affect three healthy individuals and six immunocompromised patients (Hoffmann et al., 2020; Ogundokun et al., 2020a, 2020b). The issue of COVID-19 in Pakistan arrived from the Iranian territory as several thousands of citizens travel to pilgrimage the spiritual place in Iran. After which Pakistan decided to close its border from the entry of individuals from Iran on February 23, 2020 (Lu et al., 2020). Apart from Iranian pilgrimages, several cases were traced to Afghanistan (Raza et al., 2020). Initially, the first two cases in Pakistan were announced on February 26, 2020 by the government, and it was established that the two patients had a travel history from Iran. To curtail the outbreak of the COVID-19, the federal government launched a quarantine policy on the Pak-Iran border city of Taftan (WHO, 2020a, WHO, 2020b). Up till August 5, 2020, the number of confirmed deaths cases was 6035 (GOP, 2020). The government of Pakistan has continued to enforce blended rules about social separating. Pakistan was forced to lock down mosques, huge get-togethers, mass gatherings, shopping malls, private institutions, universities, marriage halls. The government is taking strict actions and reassuring priests about the wellbeing measures. The current situation is unfavourable for Pakistanis that the cases keep increasing; therefore, specialists were encouraged to force a lockdown in numerous urban areas, yet this wasn’t easy. Many people didn’t keep to the rules of the lockdown in Italy or China, so it would be challenging to authorize in a nation like Pakistan. However, to date, partial lockdowns under section-144 have been enforced in all Pakistan (Chadsuthi et al., 2012).

Medical researchers often use linear regression to understand the relationship between drug dosage and blood pressure of patients. Quadratic regression model serves the purpose of modelling when a set of data shaped like a parabola and logarithmic regression models have been extensively used for modelling intensity of sound, yields of chemical reactions, production of goods, and growth of infants. Several statistical models can predict essential insights for public health interventions by observing “what if” scenarios. Therefore, this study aimed to predict changes in the cumulative number of COVID-19 related deaths for the coming weeks in Pakistan. This would help evaluate the impact of quarantine, social distancing, masks wearing, and smart lockdowns in the country. Three regression models were chosen, which were conventionally used in the literature for modelling and prediction purposes. Different model selection criteria have been extensively used in the literature like Kullback-Leibler divergence, Akaike information criteria, PRESS statistic, Bayesian information criteria, coefficient of determination, adjusted coefficient of determination, Mallow’s C_p_. R^2^ is one of the conventional criteria which has been used for model selection. The closer it is to 1, the better is the fit. The goodness of fit means how close an estimated value of Y is to its actual value in the given sample observations. But it increases with the increase in the predictors, so it is not the best choice because it may also increase the variance of forecast error. Adjusted R^2^ is another choice as it accommodates the problem of considerable conflict. The most reliable techniques for model selection nowadays are Akaike Information Criteria (AIC) and Bayesian Information Criteria (BIC), as it imposes a penalty for adding regressors to the model. They set a harsher punishment than R^2^ and Adjusted R^2^. The main advantage of using AIC and BIC is that they are beneficial for forecasting purposes.

## 2 Related works

Machine learning and predictive approaches have been widely applied in the earlier researches in the part of infectious ailments, which time series forecasting is a branch of. Sources include models of leptospirosis and its rainfall-temperature relationship (Chadsuthi et al., 2012), including temporal associations amid the continuing figure of cases of Plasmodium falciparum and El Niño Southern Oscillation (ENSO) (Hanf et al., 2011). Different methods have often been adopted for modelling pathogens that transpire in recurrent or repeated cycles, for instance, seasonal virus, for which a variety of researches have been released which utilized time-series demonstration to forecast possible epidemics. In (Song et al., 2016), and ARIMA (Adhikari & Agrawal, 2013) method was built to predict the regular occurrence of infection in China for 2012, whereas in (Yin et al., 2020), a predictive time series method (Tempel) was projected for influenza change estimation. Further sources include research by Lee et al. (Lee et al., 2017), who developed a time series method utilizing daily virus-linked tweet totals and used it to deliver instantaneous infection distribution evaluation. Zhang et al. (Zhang et al., 2019), designed a SARIMA method (Adhikari & Agrawal, 2013) utilizing Australian infection investigation and resident Internet pursuit data to forecast periodic flu contagion in the northern hemisphere. Time series analysis was used in (Soebiyanto et al., 2010) to examine the function of temperature variables in the public health of virus spread in 2 warm-environment areas, Hong Kong and Maricopa County (Arizona U.S.). Dominguez et al. (Dominguez et al., 1996) utilizing an alternative time series method to investigate the actions of 2 infection incidence markers in the Barcelona area to enhance their identification. As far as COVID-19 predictions are concerned, there remained a flow in the systematic research available over the preceding months. Much of these researches depend on forecasting metrics linked to coronavirus, for instance, active cases with demises in China, someplace the virus first evolved. In (Roosa et al., 2020), real-time estimates of the total sum of confirmed infected individuals were generated in the China district utilizing threedifferent phenomenological methods commonly used to predict transmittable ailments, for instance, SARS, Aids, contagion, infection, and dengue. In similar research, Yang et al. (Yang et al., 2020) merged residents relocation data and public health data to form a Susceptible – Exposed – Infectious – Removed (SEIR) method and amalgamate it with artificial intelligence system prepared on the 2003 SARS datasets to forecast China’s contagion arc. In (Li et al., 2020b),the asymmetrical feature was used to model the average and an overall number of diseases and demises, including the associated pandemic whirling opinions in China. An improved loaded auto-encoder was established in (Hu et al., 2020) to forecast the epidemic conveyance dynamics and to estimate the sum of documented COVID-19 crisis crosswise China. In contrast, Al-qaness et al. (Al-qaness et al., 2020) projected an amalgamation of an adaptive neuro-fuzzy inference system (ANFIS) and a salp-swarm-procedure-improved flower pollination algorithm (FPA) to envisage established COVID-19 crisis. Simple mean-field models were used in (Fanelli and Piazza, 2020), an analysis covering China including 2 European nations, Italy and France, to forecast the distribution of the pandemic, and most importantly the height and duration of its outbreak in both of those nations.

Cai, Jia, Feng, Li, Hsu & Lee (Cai et al., 2020) implemented the Multi-Task Gaussian Process (MTGP) regression method to boost wind speed arithmetical forecasts is studied in this article. In the proposed system, the Numerical Weather Forecasters (NWF) forecasts are first combined with a Support Vector Regressor (SVR). Pandey, Chaudhary, Gupta & Pal (Pandey et al., 2020) employed SEIR and regression models for forecast built on datasets gathered from John Hopkins University repository in India. Model efficiency was measured using RMSLE and 1.52 for the SEIR model, and 1.75 for the regression method was obtained. The fault degree of RMSLE amid the SEIR and Regression methods was 2.01. To explain the progress of the COVID-19 contagion procedure, Hou et al. (Hou et al., 2020) established a properly varied SEIR compartmentalized method. The acceptable outcomes of the properly diverse SEIR method presumed that the latent individuals’ interaction degree is amid six and eighteen, reflecting the potential effect on the disease infection rate of isolation and quarantine interventions. The findings indicate that strategies can efficiently decrease the overall sum of COVID-19 contagions and deferred the ultimate duration of diseases by decreasing the touch proportion, for instance, seclusion and confinement. Multivariate COX regression was used by Ji et al. (Ji et al., 2020)to classify the risk factors associated with development, and then implemented into the nomograph to construct an innovative estimation recording method. To test the consistency of the novel method, ROC was used (Ji et al., 2020). Hao, Xu, Hu, Wang (Hao et al., 2020) employed Elman neural network, long short-term memory (LSTM), and support vector machine (SVM). An SVM with fuzzy granulation was employed to forecast the evolution range of recently established incidents, recent demises, and recently recovered persons. To derive the association amid various features and the dispersal degree of COVID-19, Malki et al. (Malki et al., 2020) suggested different regressor machine, learning models. The machine learning procedures used in this analysis evaluate the effect on the transmission of COVID-19 of weather elements, for instance, temperature and humidity by removing the association amid the sum of reported incidence and weather elements in some provinces. In 2020, a risk model for forecasting essential diseases such as death was developed by Schalekamp et al. (Schalekamp et al., 2020) Including clinical, CXR and laboratory results. They used multivariable logistic regression. Verdict arch examination was also conducted, and a hazard simulator was imitated.

## 3 Materials and methods

### 3.1 Modelling Covid-19 death cases and functional forms

The following are the three regression models that were compared for the modeling and prediction purposes.

The linear regression model can be expressed as follow;

(1) y=a+bt+e

The logarithmic regression model has the following functional form

(2) y=a+bln(t)+e

The quadratic regression model used has the following functional form;

(3) $$\ln\;\Delta Y_{t}\;=\;a+\;b\;t+c\;t^{2}+\;e$$

Table 1 shows the estimations of parameters and value of AIC and BIC from three models for the corona deaths. R^2^ for the linear regression model is 0.928, and the Adjusted R^2^ is 0.861. The value of the coefficient of determination for logarithmic regression is 0.705, which showed that independent variables explain 70.5% of the variation in the dependent variable as compared to the R^2^ value of quadratic regression (0.997). This is much higher than logarithmic and linear regression (0.994) but does not guarantee the excellent fit of the model because as we increase the number of independent variables, the value of R^2^ changes. The essential criteria which have been extensively used in the literature for model comparison purpose are Akaike information and Bayesian information criteria. Akaike information criterion (AIC) is a fined technique based on in-sample fit to estimate the likelihood of a model to predict/evaluate the future values. AIC is an estimator of out of sample prediction error and thereby the relative quality of statistical models for a given set of data. Given a collection of models for the datasets, AIC estimates the rate of each model close to each of the other models. We ought to choose AIC and BIC criteria for the selection of a good model. The optimal model is selected based on the highest R^2^ and minimum AIC and BIC. From Table 1, it can be seen clearly that the quadratic regression model shows the best results for every model selection criterion. It has the minimum value of AIC and BIC among all three regression models, which is 330.71 and 141.81 respectively.

**Table 1:** AIC and BIC for all three regression models.

Models	Coefficients with p-value		AIC	BIC
Linear	a = -1178.407 (0.000^a^)	b = 45.685 (0.000^a^)	1537.80	672.35
Logarithmic	a = -3310.81 (0.000^a^)	b = 1280.171 (0.000^a^)	1691.10	736.85
Quadratic	a = 1.503 (0.000^a^)	b = 0.116 (0.000^a^), c = 0.000 (0.000^a^)	330.71	141.81

a Highly significant.

## 4 Results and interpretation

**Fig. 1:**
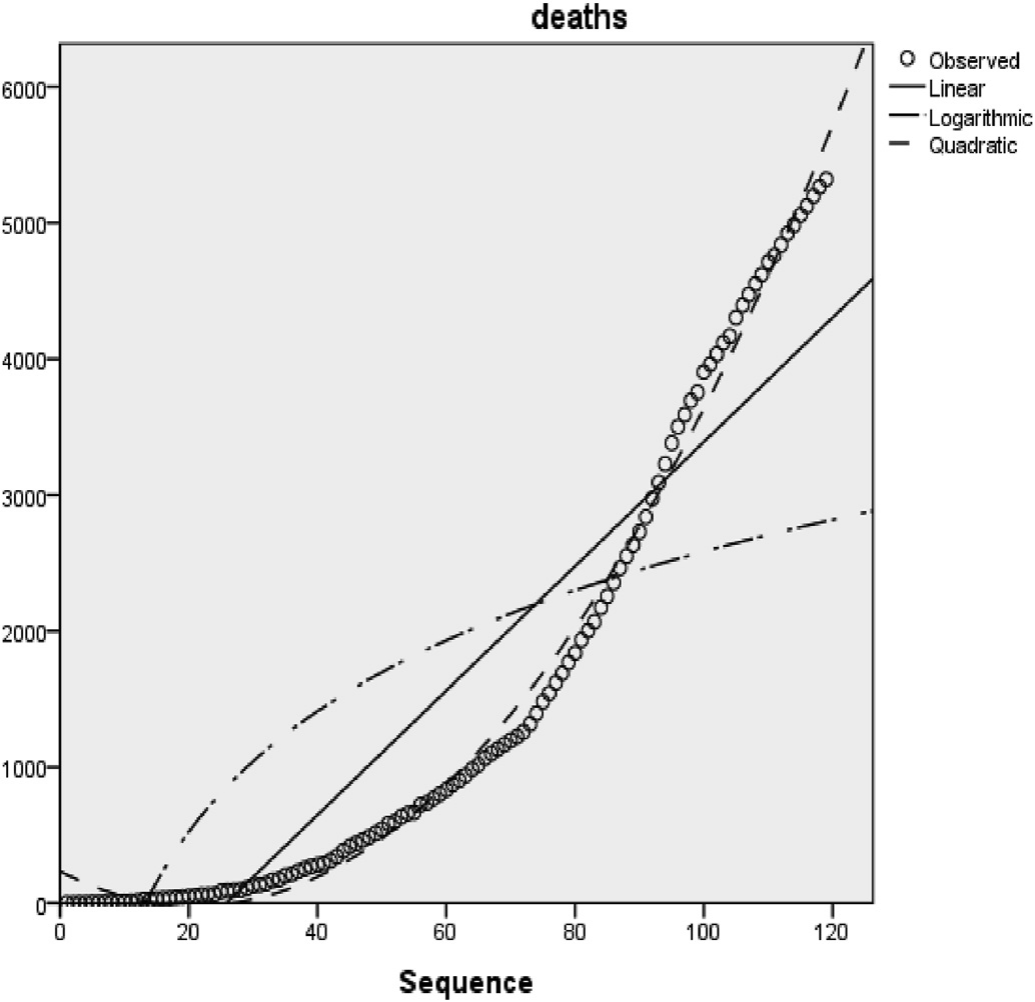
Comparison of Fitting regression models.

The value of the coefficient of determination is 0.997 showing that independent variables explain 99.7% variation in the dependent variable. The amount of adjusted R^2^ is 99.4% and also showing the same trend. The value of Durbin Watson d-statistic is 0.01, which lies in the autocorrelation area. So, there is evidence of autocorrelation, but this also does not have an impact on the prediction date. There is evidence of multicollinearity as the value of VIF is one which is showing a linear relationship between the linear and the quadratic trend, but this does not have an impact on the prediction date. The multicollinearity could have been avoided by taking the transformation of the variable. Fig. 1 shows the comparison of fitting regression models. The observed data were plotted against the fitted data of all three models. Quadratic regression shows a better fit as compared to the other two models. Fig. 3 shows the scatter plot, which indicates that there exists no precise pattern, and the points are diffused.

**Fig. 2:**
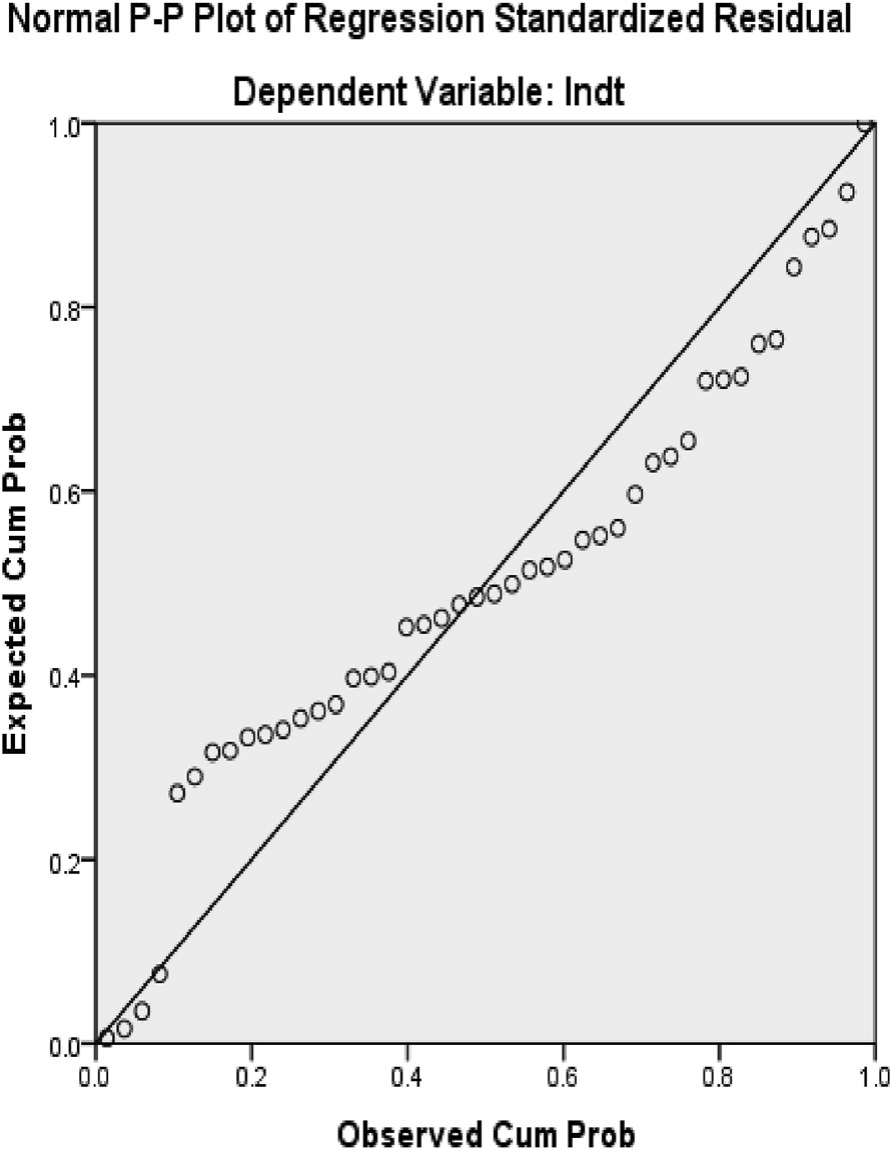
Normality plot for residuals.

For this reason, there does not exist heteroscedasticity in the quadratic regression model. Fig. 2 shows the probability plot of residuals that is meaning that the residuals follow the normal distribution. The model could be used for prediction purposes because all assumptions were met.

**Fig. 3:**
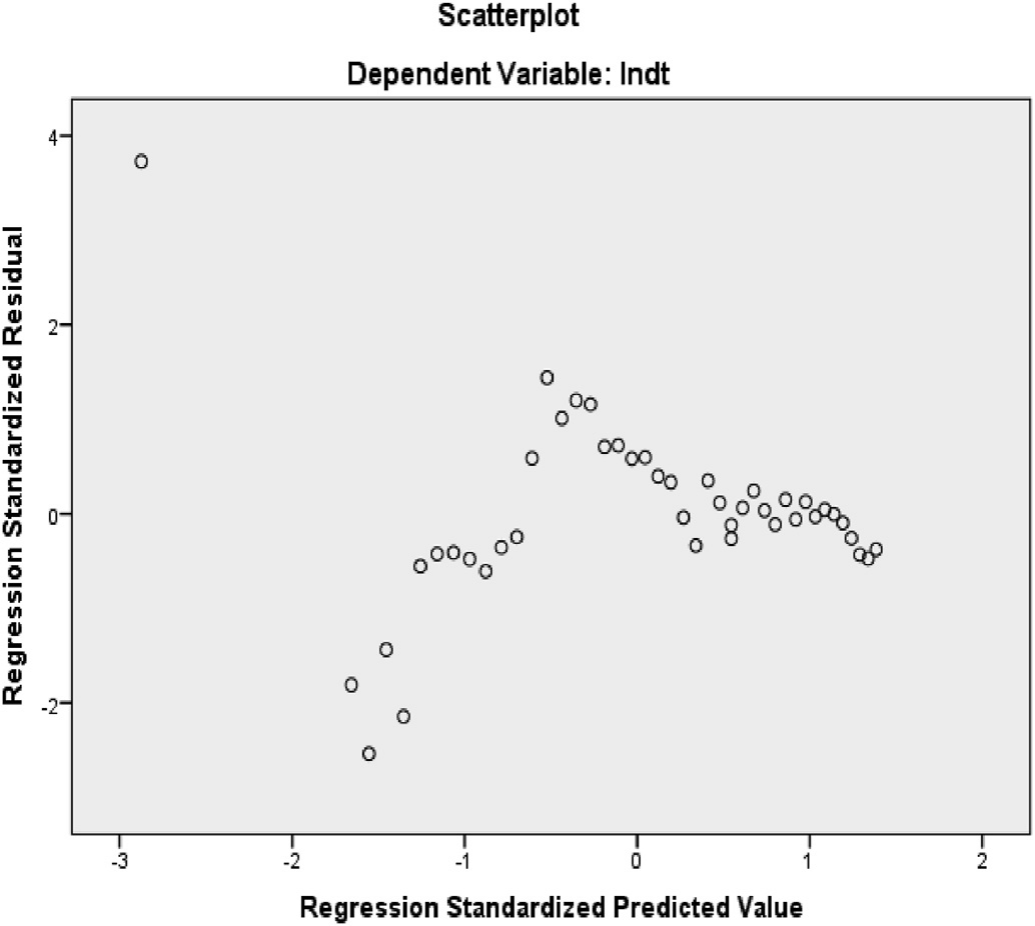
Scatter plot.

In the presentation of data, the number of deaths in Pakistan demonstrated by the was obtained from the National Institute of Health of Pakistan from February 26, 2020 up to August 5, 2020. After testing the primary unit root and some functional formats, the daily data fits well and suggests a statistically appropriate model (Raza et al., 2020). The estimations of parameters through the quadratic model for the corona deaths were mentioned in Table 2, so, the quadratic regression equation for the corona deaths is;

(4) $$\ln\;\Delta Y_{t}\;=\;1.355+\;0.124t-0.001\;t^{2}$$

**Table 2:** Coefficients of Linear and Quadratic trend with significance.

Variables	Coefficient	Standard Error	t-statistic	p-value
Intercept (a)	1.503	0.069	19.680	0.000∗∗∗
Linear Trend (b)	0.116	0.002	46.995	0.000∗∗∗
Quadratic Trend (c)	0.000	0.000	−25.965	0.000∗∗∗

The quadratic regression equation, trends with a negatively signed coefficient allows the fitted $\ln\;\Delta Y_{t}$ to reach a maximum (both local and global) and then to change its direction from increasing to decreasing. The model is relatively statistically adequate for prediction purposes. Table 3 shows the observed and fitted data of deaths cases.

**Table 3:** Observed Vs Fitted Data of Death cases.

Observed	Fitted	Observed	Fitted	Observed	Fitted	Fitted	Observed
2	5.04	292	232.59	1935	2387.54	5426	5453.38
2	5.65	312	251.09	2002	2482.4	5475	5461.02
3	6.33	343	270.8	2067	2578.62	5522	5463.47
3	7.08	385	291.79	2172	2676.06	5568	5460.85
5	7.91	417	314.12	2255	2774.54	5599	5453.1
6	8.84	440	337.83	2356	2873.97	5639	5440.25
7	9.86	462	362.99	2463	2974.17	5677	5422.27
8	10.99	486	389.66	2551	3074.97	5709	5399.33
9	12.23	514	417.9	2632	3176.2	5763	5371.43
11	13.61	544	447.76	2729	3277.68	5787	5338.66
13	15.13	585	479.31	2839	3379.22	5822	5301.15
18	16.8	599	512.59	2975	3480.63	5842	5258.91
25	18.63	636	547.67	3093	3581.73	5865	5212.11
26	20.65	659	584.61	3229	3682.33	5892	5160.92
31	22.87	667	623.45	3382	3782.16	5924	5105.38
35	25.3	724	664.24	3501	3881.08	5951	5045.74
36	27.96	737	707.05	3590	3978.82	5970	4982.07
41	30.87	770	751.91	3695	4075.22	5976	4914.62
45	34.05	803	798.85	3755	4170.04	5984	4843.53
51	37.53	834	847.93	3903	4263.05	5999	4768.99
55	41.32	873	899.19	3962	4354.04	6014	4691.18
61	45.45	903	952.64	4035	4442.76	6035	4610.31
63	49.95	939	1008.33	4118	4529.11		
66	54.84	985	1066.27	4167	4612.75		
86	60.16	1017	1126.48	4304	4693.53		
91	65.93	1067	1188.97	4395	4771.23		
93	72.18	1101	1253.76	4473	4845.67		
96	78.95	1133	1320.82	4551	4916.69		
111	86.28	1167	1390.18	4619	4984.01		
128	94.2	1197	1461.79	4712	5047.56		
135	102.75	1225	1535.67	4762	5107.12		
144	111.97	1260	1611.74	4839	5162.47		
168	121.9	1317	1690.01	4922	5213.57		
176	132.58	1395	1770.42	4983	5260.23		
201	144.07	1483	1852.91	5058	5302.32		
212	156.41	1543	1937.42	5123	5339.73		
235	169.65	1621	2023.88	5197	5372.35		
253	183.83	1688	2112.21	5270	5400.09		
269	199.01	1770	2202.33	5319	5422.92		
281	215.25	1838	2294.16	5386	5440.68		

**Fig. 4:**
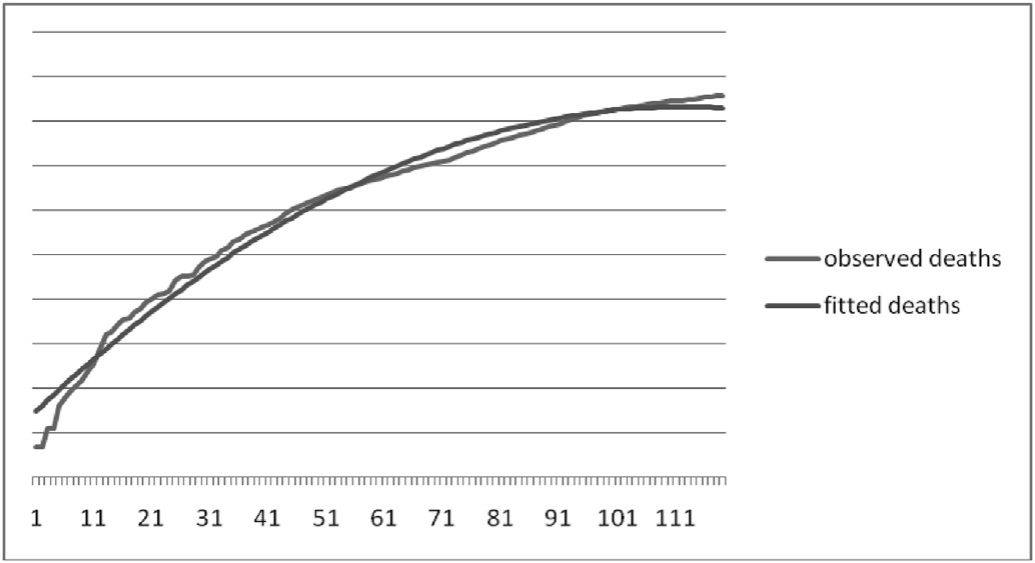
Observed VS Fitted death due to COVID-19 in Pakistan.

Fig. 4 shows the predicted and fitted deaths cases due to COVID-19 in Pakistan, which is offering a good fit for the model and suitability for prediction purposes.

## 5 Discussion

In this paper, we have proposed three regression models for the prediction of death cases by COVID-19 in Pakistan and selected quadratic modelling based on the model selection criterion. There are four stages of the epidemic, S1: exponential, S2: power law, S3: linear and S4: flat (VermaAli, 2020). The death cases in Pakistan have entered the phase of balanced and quadratic regression in this term is giving an excellent fit to the data. The same model has been used by (Tominaga, 2020) in which he showed that such a regression model is good even in the early stages of the epidemic, which is generally said to increase exponentially and monotonically. Quadratic regression modelling has also been involved in the prediction purpose for Fenton treatment of municipal landfill leachate (Lak et al., 2012). This model included both significant linear and quadratic parameters. This method of modelling has also been suggested in the situations where an estimation of the possible date of flattening the curve of the cases of infected individuals (Leon, 2020). The same modelling technique has been used for projections for first-wave COVID-19 deaths across the U.S. using social-distancing measures derived from mobile phones (Woody et al., 2020). The quadratic time trend model was also applied to the log of new cases, that accurately predict the trajectory of the epidemic in China (Linton, 2020).

## 6 Conclusion

WHO data of the whole world, together with the initial statistics about China, indicates that the daily cases and the number of patients who have been recovered from this disease are trending high. Although there are deaths because of this disease, it’s not trending upward. The number of deaths has been analyzed concerning gender, and it was concluded from the data of different countries that men are vulnerable to COVID-19 than women. This may be due to heart diseases, blood pressure, and smoking habits in men, which makes them weaker towards COVID-19 than women. It has also been observed from different countries that most affected age groups by this virus vary from country to country. The least affected age group from this virus around the globe is below 18 years. There is a significant difference between the average deaths and recovery cases since the recovered patients are right in numbers than fatalities. Studies showed that as far as issues and death rates are concerned, age and gender impacted differently.

The Quadratic regression model has been selected from three regression models based on the model selection criteria; conventionally used methods are AIC and BIC for discussing the death cases. The model which has the smallest value of AIC and BIC among all the regression models, that model is used for modelling and prediction. After applying the predictive model, the rate of mortality is predicted to decrease by the end of October. The total number of deaths will be reached at the maximum point; then, it will gradually decrease. This indicates that the curve of total deaths will continue to be flat, i.e., it will shift into a constant that is also the upper bound of the underlying function of total deaths. For the deterministic part of the model, the definition remains. This interpretation holds for the deterministic aspect of the model. The coronavirus carriers are anonymous, and everyone is a potential carrier of the virus that could cause great havoc to society. The outbreak may rise to an unmanageable scenario. With the increased number of deaths, the government should consider lockdown decision with strict rules and regulations as well as the public should follow simple and basic prevention guidelines.

## Declaration of competing interest

The authors declared there is no conflict of interest during this study.
